# Successful Management of Intraprocedural Arterial Dissection During Carotid Artery Stenting With an Overlapping CASPER Rx Stent for Tortuous Vessel: A Case Report

**DOI:** 10.7759/cureus.74475

**Published:** 2024-11-26

**Authors:** Takeshi Miyata, Yoshitaka Tsujimoto, Takenori Ogura, Yuji Agawa, Taketo Hatano

**Affiliations:** 1 Neurosurgery, Kokura Memorial Hospital, Kitakyushu, JPN

**Keywords:** arterial dissection, carotid artery stenosis, carotid artery stenting, casper stent, stent resheath

## Abstract

Carotid artery stenosis is a significant cause of ischemic stroke, often necessitating interventions like carotid artery stenting (CAS) to restore adequate blood flow. However, complications like intraprocedural arterial dissection can arise during the procedure. This report presents a case of intraprocedural arterial dissection during CAS using a CASPER Rx stent. A 77-year-old male, previously diagnosed with asymptomatic right internal carotid artery (ICA) stenosis two years earlier, was presented to our institution with transient left upper limb paralysis. CAS with a CASPER Rx stent was planned due to the progression of symptomatic ICA stenosis. During the procedure, an arterial dissection occurred in the vessel wall just distal to the stent but was successfully managed with additional stenting. The patient experienced no postoperative neurological deficits and was discharged in stable condition. This case highlights the importance of careful resheathing to avoid unintended advancement of the stent system, particularly in cases of ICA stenosis with severe vessel tortuosity. Furthermore, the present case also emphasizes the critical need of timely intervention to prevent serious complications.

## Introduction

Carotid artery stenosis is a prevalent cause of ischemic stroke, accounting for approximately 10-20% of cases [1]. Carotid artery stenting (CAS) has emerged as a less invasive alternative to carotid endarterectomy (CEA) for treating internal carotid artery (ICA) and/or common carotid artery (CCA) stenosis [1-4]. However, recent large-scale clinical studies have identified various risk factors associated with complications during CAS, including both anatomical factors (e.g., ICA-CCA angulation, plaque characteristics) and technical factors (e.g., type III aortic arch, cerebral protection device, and closed-cell stent) [2-5]. Among these complications, intraprocedural arterial dissection has been highlighted as a significant risk [6-8].

The CASPER Rx stent (Terumo, Tokyo, Japan), a dual-layered bladed nitinol stent designed to minimize kinking and enhance conformability to complex arterial anatomies, has gained widespread usage in CAS procedures [9-11]. Its design reduces the risk of embolization by providing superior scaffolding and minimizing plaque prolapse [12]. A notable feature of the CASPER Rx stent is its repositionability during deployment, allowing operators to adjust the stent’s position within the catheter up until 50% of the stent which has been deployed [13-15]. Despite these advantages, complications such as postoperative ischemic infarction and arterial dissection can occur. Arterial dissection, in particular, may arise due to mechanical stress on the arterial wall during stent deployment or balloon dilation, especially in cases involving tortuous or heavily calcified vessels [16]. Recognizing and managing these complications promptly during the procedure is crucial to prevent serious outcomes, including postoperative ischemic stroke.

In this report, we present a case of significant ICA stenosis complicated by severe arterial tortuosity. During the CAS procedure using the CASPER Rx stent, an arterial dissection occurred while resheathing and repositioning the stent. This complication was successfully managed through the deployment of an additional overlapping CASPER Rx stent. This case underscores the importance of identifying and minimizing procedural risks, particularly in patients with complex vascular anatomy, and emphasizes the critical need for timely intervention to prevent further complications.

## Case presentation

**Figure 1:**
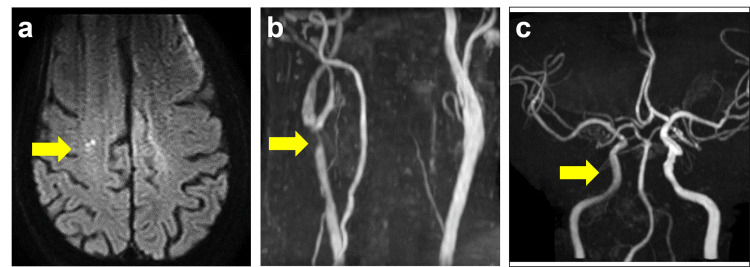
Preoperative images a) Head MRI diffusion-weighted image shows an acute cerebral infarction in the right frontal subcortical white matter (arrow). b) Cervical MRA demonstrates severe stenosis of the right intracranial carotid artery (ICA) (arrow). c) Head MRA shows poor visualization of the right ICA and middle cerebral arteries (arrow).

A 77-year-old male, previously diagnosed with asymptomatic right internal carotid artery (ICA) stenosis two years prior, was presented to our institution with transient left upper limb paralysis. His past medical history was significant for cardiac arrest due to ventricular fibrillation, an old myocardial infarction, and severe tricuspid valve regurgitation. Upon admission, the patient was alert, fully ambulatory, and exhibited no motor deficits. Radiological imaging, including head magnetic resonance imaging (MRI) and carotid ultrasonography (CUS), revealed a high-grade stenosis in the right ICA, with evidence of new microinfarcts in the right hemisphere (Figure 1a-1c). CUS demonstrated a peak systolic velocity of 290 cm/s in the right ICA, indicative of significant hemodynamic compromise.

**Figure 2:**
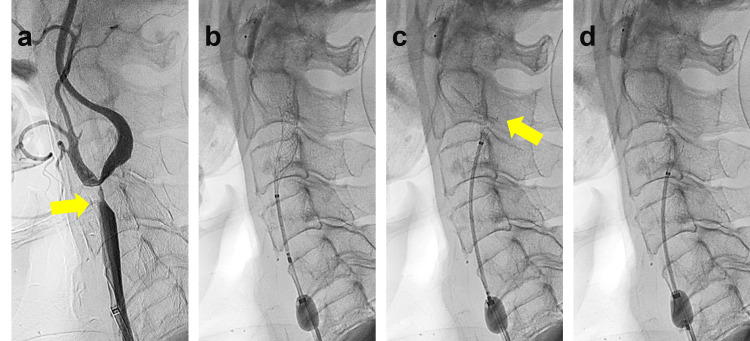
Intraoperative findings a) Cervical digital subtraction angiography (DSA) demonstrates severe stenosis of the right internal carotid artery (ICA) (arrow) and reveals a marked angulation of the ICA, bending backward before turning forward. b) Initial deployment of a CASPER Rx stent following distal filter protection and balloon angioplasty. c) Resheathing of the CASPER Rx stent to adjust its position. Note the potential risk of vessel wall damage at the outer curve of the right ICA (arrow) caused by the stent's distal edge. d) The replacement of a CASPER Rx stent at the proximal ICA.

**Figure 3:**
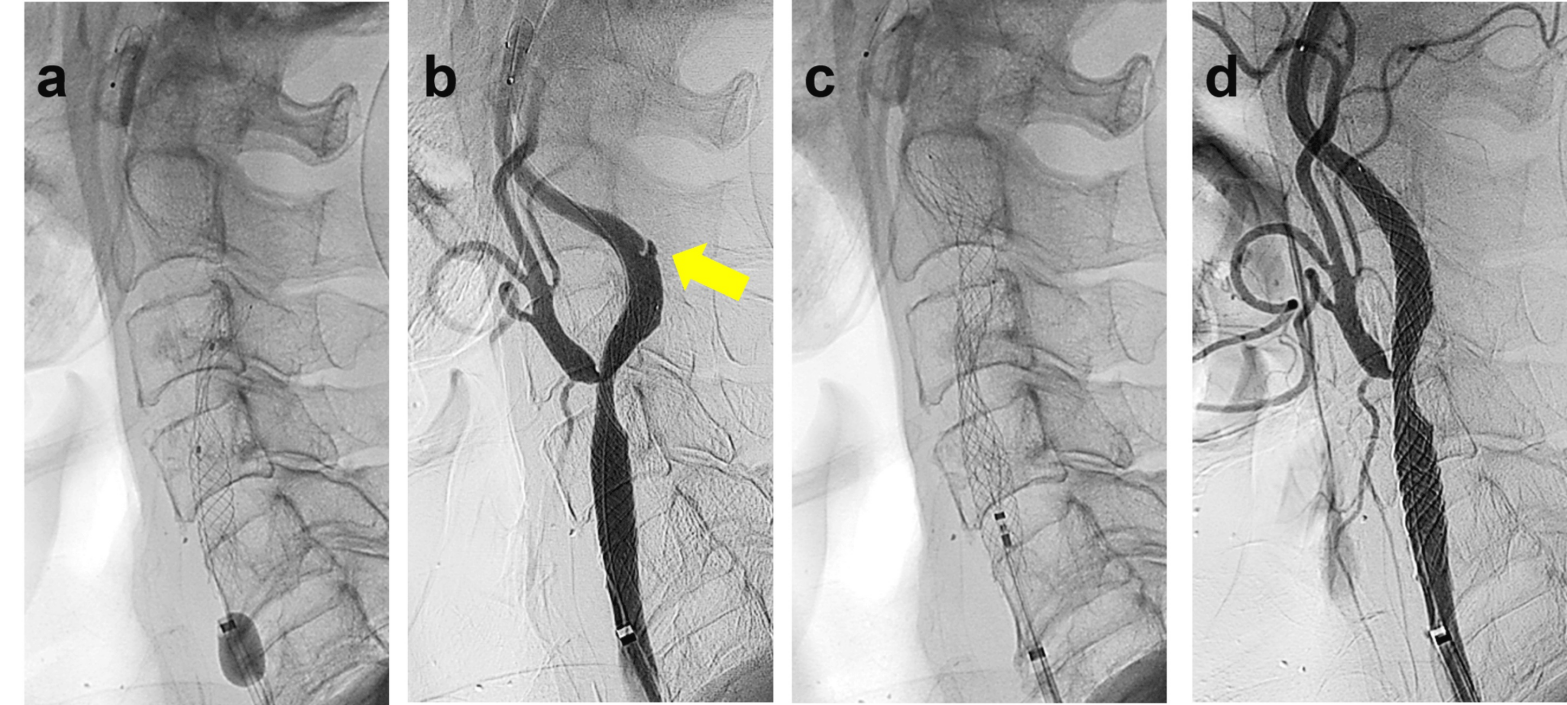
Intraoperative findings following intraprocedural dissection a) Balloon angioplasty post-stenting. b) Cervical digital subtraction angiography (DSA) reveals an arterial dissection at the outer curve of the right ICA, adjacent to the stent's distal edge, where initial stent placement was attempted. c) An additional CASPER stent was deployed to overlap and fully cover the dissection site. d) Cervical DSA confirms favorable dilation of the right carotid lumen, with no abnormalities detected.

Given the progression of the ICA stenosis and the occurrence of recent ischemic events, CAS was planned under local anesthesia via the right femoral artery. The procedure was scheduled two weeks after the initiation of dual antiplatelet therapy with aspirin and clopidogrel. After sheath placement, an 8Fr Optimo balloon guide catheter (Tokai Medical Products, Aichi, Japan) was introduced to the right CCA, and angiography confirmed high-grade ICA stenosis (Figure 2a). With proximal flow control, a SpiderFX distal protection device (Covidien, Mansfield, MA, USA) was successfully deployed in the distal cervical segment of ICA. Pre-stent angioplasty was then performed using a Coyote 3.0×40mm balloon dilatation catheter (Boston Scientific, Marlborough, MA, USA). A CASPER Rx 8mm x 25mm stent was deployed, but it became slightly displaced due to the vessel’s tortuosity (Figure 2b). The stent delivery system shifted just distally within the ICA, requiring resheathing and redeployment of the stent (Figure 2c, 2d; Figure 3a).

**Figure 4:**
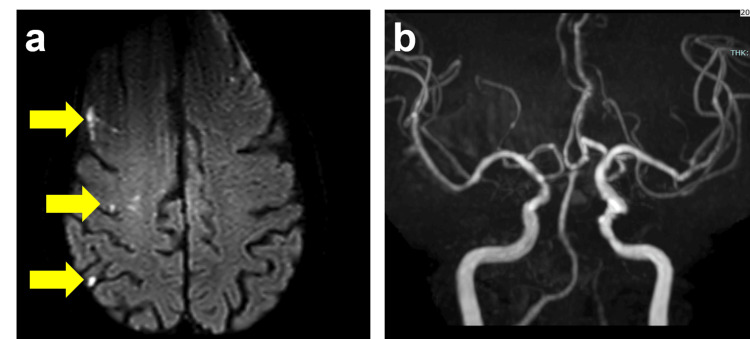
Postoperative findings a) Head MRI diffusion-weighted image reveals several asymptomatic acute cerebral infarctions in the right frontal and parietal subcortical white matter (arrows). b) Head MRA shows improved visualization of the right internal carotid artery and middle cerebral arteries.

Angiography revealed an irregularity in the vessel wall of the ICA just distal to the stent, suggesting an arterial dissection (Figure 3b). Despite multiple repeated angiographic assessments over 30 minutes, no improvement was observed. Consequently, an overlapping CASPER Rx 8mm x 30mm stent was deployed (Figure 3c), effectively covering the dissected segment. Post-stent angioplasty was performed using a Sterling 4.0 × 20mm balloon dilation catheter, inflated for 15 seconds at 8 atm, restoring luminal integrity (Figure 3d). The patient remained neurologically stable throughout the procedure (Figure 4a, 4b). The postoperative course was uneventful, and the patient was discharged five days later without any neurological deficits. During the three-year postoperative follow-up period, no imaging evidence of dissection progression or recurrent stenosis was observed.

## Discussion

We report a case of intraprocedural arterial dissection during CAS using a CASPER Rx stent. This case highlights the potential risk of arterial dissection during resheathing, particularly in cases of ICA stenosis with increased vessel tortuosity, where unintended movement of the stent system can lead to vascular injury.

CAS has become a widely accepted treatment for ICA stenosis, especially in patients at high risk for CEA [1-4]. New-generation dual-layer nitinol stents, such as the CASPER Rx stent, have been reported to significantly reduce atherosclerotic plaque prolapse and minimize adverse neurologic events, offering greater flexibility during CAS procedures [9-11]. The micromesh design of the CASPER stent enables it to trap and exclude thrombus and plaque debris, helping to prevent embolic events originating from the stenotic lesion [12-15]. However, despite these advantages, dual-layer stents do not completely eliminate the risk of intraprocedural vessel injury. Complications such as arterial dissection during CAS with a CASPER Rx stent, particularly in anatomically complex cases, remain incompletely understood and warrant further investigation.

**Figure 5:**
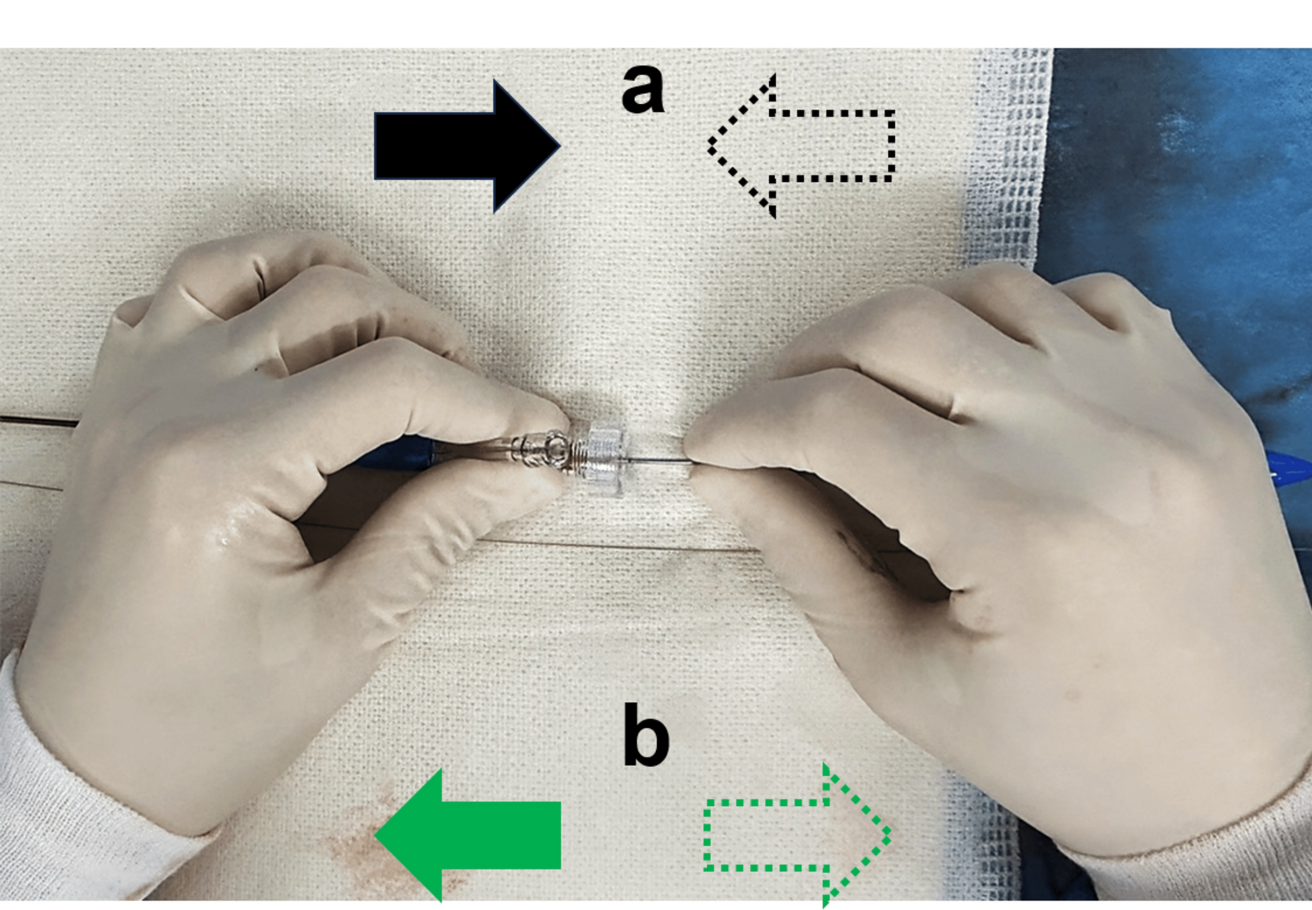
Hand movements of the operator during CASPER Rx stent deployment and resheathing a) The operator should stabilize the inner shaft of the CASPER Rx stent delivery system with the right hand, ensuring no unintended proximal movement when pulling the outer sheath with the left hand. b) The operator should stabilize the inner shaft of the CASPER Rx stent delivery system with the right hand to prevent distal movement while resheathing the outer sheath with the left hand.

Intraprocedural arterial dissection during CAS is a rare but serious complication that can lead to adverse outcomes such as acute carotid stent thrombosis or stroke [6,8,16]. Risk factors for dissection include anatomical challenges such as increased vessel tortuosity, the degree of stenosis, and the presence of calcified plaques [16]. Even if recent studies have reported varying rates of complications associated with CAS [2-5,17], the International Carotid Stenting Study documented a 7.4% incidence of stroke, myocardial infarction, or death within 30 days of CAS, associated with factors such as age, vessel tortuosity, and stent type influencing outcomes [3]. Additionally, the use of distal protection devices like SpiderFX can reduce the risk of embolization [4,16], but may not prevent direct mechanical complications like arterial dissection. Although there were few reports associated with anatomical factors and iatrogenic dissection during CAS procedure, Ito et al. recently reported a case of delayed iatrogenic dissection by CAS for ICA stenosis with severe vessel tortuosity [8]. According to the user’s instructions of CASPER Rx stent, the operator should stabilize the inner shaft of the CASPER Rx stent delivery system with the right hand, ensuring no unintended proximal movement when pulling the outer sheath with the left hand (Figure 5a) [18]. Furthermore, the operator also should stabilize the inner shaft of the CASPER Rx stent delivery system with the right hand to prevent distal movement while resheathing the outer sheath with the left hand (Figure 5b) [18]. In this case, it was thought that the stent was unexpectedly advanced and the distal edge of the stent damaged the vessel wall of the right ICA because of severe vessel tortuosity in resheathing the outer-sheath with the left hand.

It still remains unelucidated whether stenting for intracranial or extracranial carotid artery dissection is more effective compared to conservative medical treatment with antiplatelet agents [19-22]. Iatrogenic dissection of the cervical arteries is a well-known complication of cerebral angiography or interventional radiological procedure, with a reported incidence of 0.15-0.6% [6,8,16,23]. Although most cases of iatrogenic dissection were recently reported to be safely managed without further intervention in the acute setting, cerebrovascular dissections have been known to have the potential to cause infarction of the brain via direct vessel occlusion or as a source of thromboembolic material [6]. Recent numerous reports revealed the effectiveness of the treatment of intracranial or extracranial dissections with stents [7,8,24,25]. Borota et al. reported their experience in the treatment of iatrogenic dissections of cervical arteries with the Neuroform Atlas stent (Stryker Neurovascular, Fremont, CA, USA) during endovascular treatment [7]. In this case, since multiple repeated angiographic assessments over 30 minutes could not reveal any improvement of the dissection, additional overlapping stenting was planned by a CASPER Rx stent as on-label use. The prompt recognition and management of the dissection might lead to the prevention of more severe complications. Further research will be needed to determine how intraprocedural iatrogenic dissection during the CAS procedure is required for additional stenting.

## Conclusions

This report presents a case of significant ICA stenosis complicated by severe arterial tortuosity. During CAS with the CASPER Rx stent, an arterial dissection occurred while resheathing and repositioning the stent. However, this complication was successfully managed by deploying an additional overlapping CASPER Rx stent, thanks to prompt recognition and timely intervention. This case highlights the importance of careful resheathing without unintended advancement of the stent system, particularly in cases of ICA stenosis with severe vessel tortuosity. Meticulous attention during the procedure can help prevent intraprocedural arterial dissection. Furthermore, the present case underscores the need to recognize and minimize procedural risks, especially in patients with complex vascular anatomy, and emphasizes the critical importance of timely intervention to avoid serious complications.
